# Highly Conductive Carbon Nanotube-Thermoplastic Polyurethane Nanocomposite for Smart Clothing Applications and Beyond

**DOI:** 10.3390/nano9091287

**Published:** 2019-09-09

**Authors:** Sandra Lepak-Kuc, Bartłomiej Podsiadły, Andrzej Skalski, Daniel Janczak, Małgorzata Jakubowska, Agnieszka Lekawa-Raus

**Affiliations:** Faculty of Mechatronics, Warsaw University of Technology, 00-661 Warsaw, Poland (S.L.-K.) (B.P.) (A.S.) (D.J.) (M.J.)

**Keywords:** carbon nanotubes, CNT–polymer composite, nanocomposite fibers, smart clothing, printed electronics, structural electronics

## Abstract

The following paper presents a simple, inexpensive and scalable method of production of carbon nanotube-polyurethane elastomer composite. The new method enables the formation of fibers with 40% *w*/*w* of nanotubes in a polymer. Thanks to the 8 times higher content of nanotubes than previously reported for such composites, over an order of magnitude higher electrical conductivity is also observed. The composite fibers are highly elastic and both their electrical and mechanical properties may be easily controlled by changing the nanotubes content in the composite. It is shown that these composite fibers may be easily integrated with traditional textiles by sewing or ironing. However, taking into account their light-weight, high conductivity, flexibility and easiness of molding it may be expected that their potential applications are not limited to the smart textiles industry.

## 1. Introduction

The area of smart or intelligent textiles/clothing integrating textiles with electronics is currently one of the most invested and rapidly developing modern technology fields [1,2,3,4,5,6,7]. Smart clothing can serve not only an aesthetic purpose but first and foremost improve our living standards, safety and health, e.g., by monitoring vital signs and environment parameters, facilitating communication, or producing energy [8,9,10,11]. Taking into account a great number of potential solutions which could be enabled by smart clothing technologies, it is highly desirable to conduct research on new electronics, materials and manufacturing methods which may serve the development of novel e-textile applications.

Particularly important in this respect is the development of electrically conductive and electronically active materials fulfilling additional requirements of the textile industry, such as flexibility, elasticity, strength, light-weight, washability and low-cost of production. A very interesting group of materials here are polymer nanocomposite fibers with carbon nanotubes used as electrically conductive nanofillers. Carbon nanotubes (CNTs) are light-weight highly conductive, high aspect-ratio pure carbon allotropes which may be already produced on an industrial scale and at low cost.

In the early days of research on CNTs the production of polymer composite fibers was one of the major fields of study [12,13,14,15,16,17]. These fibers reached respectably high conductivities of up to 10 S/cm in the non-annealed and non-doped state and 5 times higher upon doping of CNTs [12,15]. Unfortunately, the choice of polymers used at that time was quite limited and often not oriented on the textile industry.

This research area was soon taken over by the development of pure CNT fibers [18]. Although these fibers have already exceeded the MS/m conductivities, they are not very elastic and cannot be processed like polymer based materials [19,20,21]. The pure CNT fibers currently available on the market are also very expensive. For these reasons the research in the area of CNT composite fibers for smart textile applications is still highly desirable and continued.

The polymer matrix which would fulfil the requirement of elasticity, easiness of processing, price and usability for the textile industry, is thermoplastic polyurethane (TPU). Up to now, there have been several reports showing the production of CNT-TPU composite fibers [22,23,24,25,26]. However, these composites were all characterized by low electrical conductivity of up to several S/m. This issue was mainly due to a very low content of CNTs in the composite originating from the ineffective methods of composite production, based on direct mixing of polymer melt/solution with the CNT powders.

To address this issue, in the following work we developed a simple and low-cost method of production of highly conductive, flexible and light-weight composites fibers, which are composed of well-known to textile industry thermoplastic polyurethane (TPU) and carbon nanotubes (CNTs). The method enables precise control of electrical and mechanical properties of the composites by optimization of the nanofiller content. The as-produced fibers may be sewn into fabrics or molded/ironed and used in textile printed designs. Finally, it is expected that such composites may be also used beyond the textile industry e.g., as filaments for 3D printers.

## 2. Materials and Methods

### 2.1. Materials

The materials used for the manufacture of the composites included industrial grade carbon nanotubes purchased from Nanocyl SA., Belgium and thermoplastic polyurethane obtained from BASF Corporation, Germany product Elastollan^®^ 1170 A 10,000. The manufacture process involved also the use of tetrahydrofuran (THF) purchased from Linegal Chemicals, Poland and of surfactant AKM-0531 obtained from NOF Corporation, Japan.

### 2.2. Composite Preparation

**Figure 1:**
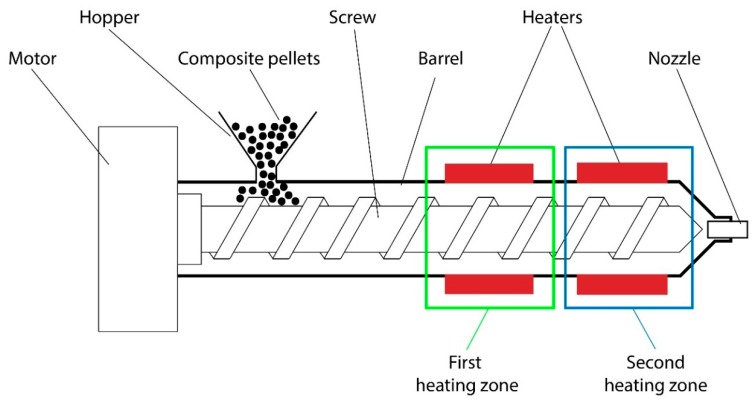
Schematic diagram of a purpose designed screw extruder used for the composite fiber preparation.

Ultrasound mixing was performed using sonics vibra-cell VCX 750 sonicator with 70% of nominal power. Mixing of the CNT/THF solution with polymer was performed using Heidolph MR-HEI Standard magnetic stirrer. The fiber was formed using a purpose designed screw extruder, equipped with two independently regulated heating zones, as presented in Figure 1. In this system, the speed of the screw is constant and set at 38 rpm, while the heating zones temperatures may be adjusted in the range from 25 to 500 °C. The system enables also the change of nozzle diameters.

### 2.3. Composite Analysis and Testing

The resistance of the fibers was measured using the True RMS multimeter UT804, Uni-Trend Technology Co., Ltd., China. The two-point probe method was used for all the measurements. Connections between fibers and electrodes were made with the aid of organic solvent based commercial Silver Conductive Paint Electrolube, United Kingdom. Current carrying capacity of the fibers was measured using a dedicated setup containing a DC power supply QL564P, Aim TTi, United Kingdom and a DC Keithley 2000 multimeter, Keithley Instruments, Cleveland, Ohio, USA all controlled via LabVIEW software, National Instruments Corporation, Austin, Texas, USA. Tensile tests were performed using QC-506B1 instrument, Cometech Testing Machines Co., Ltd., Taiwan. The gauge length for all tests was set to 20 mm and the testing speed to 2 mm·min^−1^. Weighing was performed on Precisa 125 A precision balance, Precisa Gravimetrics AG, Switzerland.

The Raman scattering spectra of fibers were measured on a LabRam 300 spectrometer, with a 17 mW 633 nm red laser. LabSpec 5 software (Horiba UK Ltd., United Kingdom) was used to control the laser operation and process of data collection as well as to generate the images.

Scanning electron microscope images were performed using SU 8000 SEM, Hitachi, Japan working in a secondary electron mode.

## 3. Results and Discussion

### 3.1. Material Production Method

As mentioned in the introduction, the production methods used for the fabrication of pure CNT-TPU composite fiber reported so far, were based on simple mixing of polymer melt/solution with the nanofiller powder which resulted in low nanofiller content and therefore low conductivity [22,23,25,26]. So as to solve this issue, in the following paper, a different method was proposed. A weighed amount of CNTs was sonicated for two hours in excess amount of tetrahydrofuran (THF) (CNTs concentration was not higher than 2 wt%). A 2 wt% of surfactant in relation to nanocarbon mass, was also added to the solution, so as to facilitate deagglomeration and suspension of the CNTs. Subsequently, the weighed amount of TPU was mixed with the solution for five hours at 50 °C. Afterwards, the samples were left under the fume hood for 48 h to evaporate the solvent. Thus, the final content of carbon nanotubes in the composite was controlled only by the weight ratio of CNTs and polymer added to the solvent. Taking into account an extremely small amount of surfactant, this assumption should not introduce any significant error.

After evaporation of the solvent, the composite was palletized and formed into a fiber using a hot mixing extrusion process. So as to obtain comparable results, the nozzle of 1.5 mm diameter was used for the production of all composites. The regulated temperatures of the heating zones were set at 140 ℃ and 160 °C, for the first and second heating zone, respectively (Figure 1). Lowering of the temperatures resulted in insufficient plasticization of the matrix material, which hindered the extrusion process and made the material inhomogeneous. On the other hand, too high temperatures resulted in thermal degradation of the polymeric matrix. The optimization of the fiber extrusion process enabled a continuous formation of the highly homogeneous fibers of any length and high diameter uniformity along the length.

### 3.2. Composite Characterisation

The composite production method proposed above enabled the formation of a composite fiber with a much higher content of carbon nanotubes than reported so far. The maximum weight percent of carbon nanotubes amounted to 40% as compared to 5% *w*/*w* of CNT reported previously [22,25,26]. Further, the increase in the content of nanotubes in the fiber was associated with a visible deterioration of elastic properties. At 50% *w*/*w* of nanotubes, the fiber crumbled and it was not possible to extrude it continuously.

**Figure 2:**
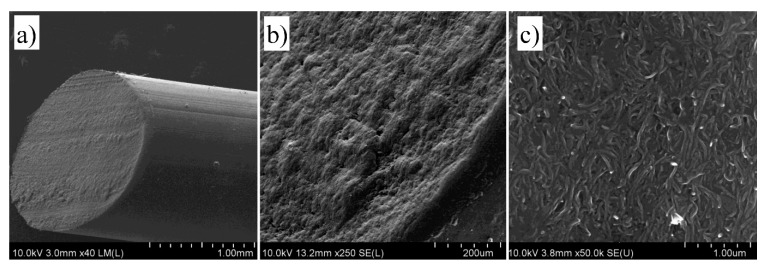
(**a**–**c**) Scanning electron microscope images of cross-section of CNT/TPU composite fiber performed at increasing magnifications.

Figure 2 presents scanning electron microscope images performed on the cross-section of the sample with 40% *w*/*w* of CNT in TPU. The images show high isotropy of the fiber structure at the microscale (Figure 2a,b) as well as clear presence of carbon nanotubes (Figure 2c) at the nanoscale. It is also visible that CNTs are very uniformly distributed in the polymer matrix.

**Figure 3:**
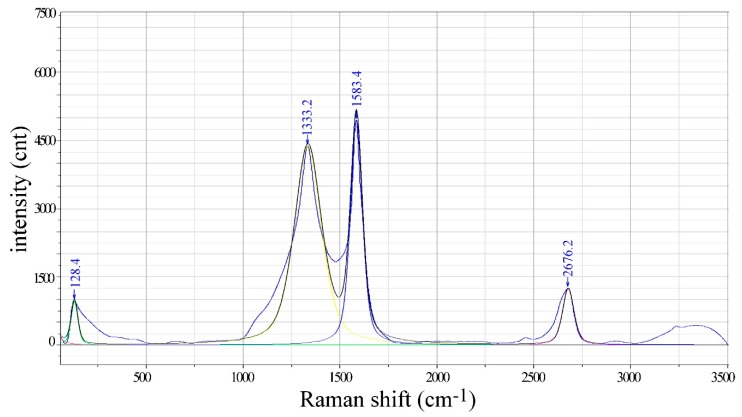
Raman spectrum for 40% *w*/*w* CNT in TPU composite fiber.

The same material was also subjected to Raman spectroscopy which is an analytical tool widely used for the characterization of carbon nanotubes (Figure 3). The characteristic features of CNT Raman spectra are: RBM (radial breathing mode), D-band, G-band, and 2D (or G’)-bands. RBM peaks appear at low wavenumbers and are visible only for a high concentration of single wall carbon nanotubes (SWCNT). D-band observed at approximately 1340 cm^−1^ is associated with the presence of disordered and amorphous carbon [27]. G-band appears around ~1580 cm^−1^ and is related to in-plane carbon-carbon bond stretching [28]. Last feature characteristic for CNT materials is an overtone to D-feature, known as 2D-band or G’-band. This is a peak observed for all sp^2^ bonded carbons, qualitatively not related to structure disorder [29]. Finally, the disorder and impurity of the CNT materials are often assessed based on intensity ratio for and D and G peak I_D_/I_G_. The smaller it is the better the quality of the material.

The analysis of the Raman spectroscopy results presented in Figure 3, shows that all features characteristic for CNT materials are present in the CNT-TPU spectrum, indicating a clear presence of these materials. It is quite interesting to find high intensity RBM peaks testifying a presence of SWCNTs in the industrial grade CNTs batch used for the manufacture of the fibers. Finally, it is worth mentioning that D-band is broad and I_D_/I_G_ intensity ratio is high, which could indicate low graphitization and purity of the material. However, taking into account that all CNTs are coated with TPU it should be rather understood as a feature characteristic for the composite.

The as-produced nanotube-polymer fibers have been further subjected to the tests of electrical conductivity, current carrying capacity and stress-strain tensile tests. So as to understand the influence of the CNT content on the properties of the fibers. The results obtained for the composite composition containing 40% *w*/*w* of carbon nanotubes were also compared to the results obtained for fiber containing 20% *w*/*w* of CNT in TPU.

The electrical conductivity of the 40 wt% CNT sample amounted to 671 ± 22 S/m. This is over an order of magnitude better result than for previously reported CNT/TPU fibers. This is also a comparable result to other non-doped and non-annealed CNT-polymer composite fibers [12]. Decrease in a CNTs content to 20 wt%, results in a drop of conductivity by over 2 orders of magnitude to 4.2 ± 0.7 S/m. However, this is still a very good result as compared to other CNT/TPU fibers.

**Figure 4:**
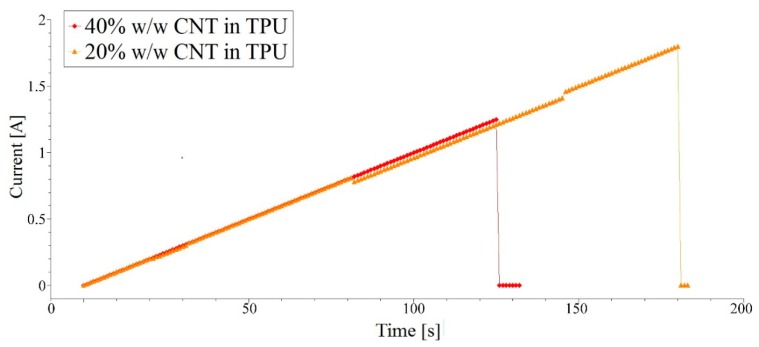
Measurements of the current carrying capacity of composite fibers, with 20% *w*/*w* and 40% *w*/*w* content of CNTs.

Figure 4 presents the results of current carrying capacity tests, performed in a step mode i.e., DC current has been increased by 0.01 A every 1 s. It is visible that for the 40% *w*/*w* content of nanotubes, the maximum current reached 1.25 A, while for 20% *w*/*w* 1.8 A. This result is quite unexpected taking into account the conductivities of the fibers. However, it is possible that the differences in the density of the materials and different heat removal conditions are responsible for such discrepancies.

Nevertheless, it should be rather noticed here that both fibers have shown very good results and failed at over 1 A. Taking into account poor thermal and electrical conductivity of TPU it is a very impressive outcome enabling many electronic applications.

**Figure 5:**
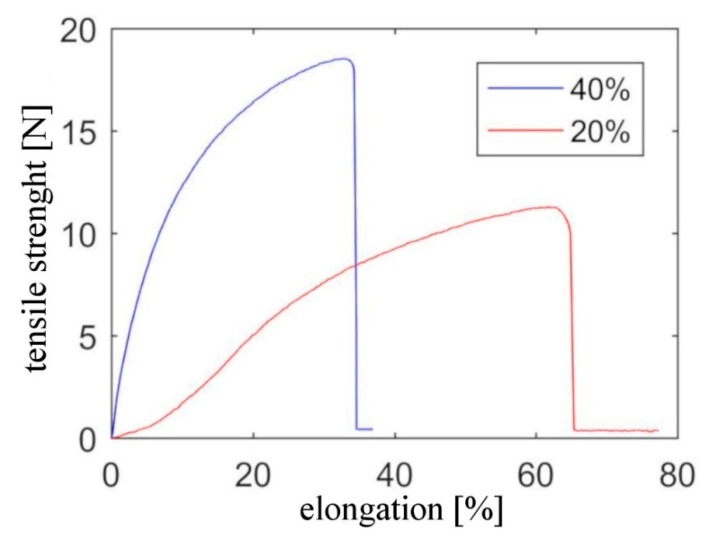
Stress and strain curves of composite fibers containing 20% and 40% *w*/*w* of nanotubes.

Finally, stress-strain tests performed on both types of samples presented in Figure 5 demonstrate that the fibers show classical elastic and plastic deformation regions observed for pure CNT fibers and other CNT-polymer composite fibers [21,30,31]. However, what is more important mechanical properties of the fibers may be easily controlled by changing the CNT content in the composite. Clearly, the 40 wt% composite shows higher strength and lower maximum elongation, while the decrease in strength is correlated with a higher elasticity of the 20 wt% fibers.

It is also worth noting that the maximum elongations of 35% for 40% *w*/*w* CNT in TPU and 70% for 20% *w*/*w* CNT in TPU are very high as for CNT materials [30,31]. This property should be also particularly useful in textile applications.

Finally, the proposed composites are also very lightweight as the density of the composite fibers amounts to 1.1 ± 0.1 g/cc only. The above presented analysis shows that using the proposed method of CNT-TPU fiber manufacture it is possible to produce highly conductive light-weight composite materials with the potential to be applied in modern electronics including smart textiles.

### 3.3. Application

**Figure 6:**
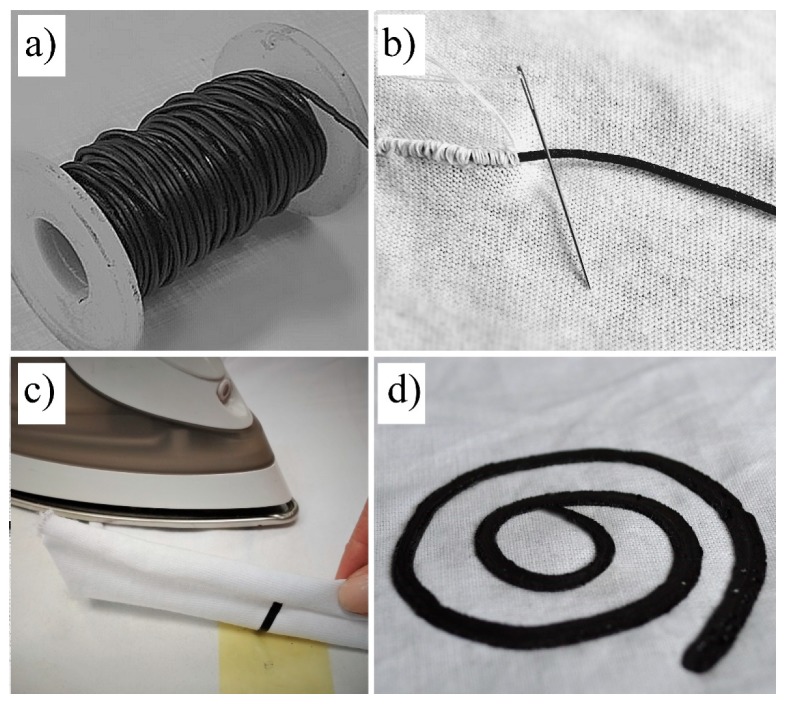
Composite CNT/TPU fiber: (**a**) Wound on a reel; (**b**) sewn into fabric; (**c**) and (**d**) ironed onto fabric.

Application of the composite in any electronics area requires separate extensive research. Taking into account that the produced composites may be particularly interesting for smart textiles applications we approached this area as an example. Firstly, 10-metres-long composite with 40% *w*/*w* of CNTs has been manufactured and wound on a reel (Figure 6a). Secondly, the issue of introduction of such composites into the fabrics, has been considered.

The revision of the literature indicates that there are many methods by which the conductive elements may be integrated into the fabrics. However, they may be generally divided into two main types which include weaving/sewing or depositing of the coating layer on the fabric [6,7,32,33]. In the case of our CNT-TPU composites both methods are possible.

As shown in Figure 6b the self-standing composite fibers were both sewn into a fabric and transferred onto fabric by a thermal process, where the polymer softens and attaches to the woven classical yarns. Such a “textile print” was performed using a simple iron as shown in Figure 6c,d. The integrated fibers are very strongly attached to the fabric (cannot be removed by hand), while fatigue testing showed that a bending of the material 100 times by at least 170° did not cause any mechanical damage to the conductive pathway or change in electrical conductivity.

## 4. Conclusions

In this paper a new method of the production of composite carbon nanotubes/thermoplastic polyurethane fibers has been presented. The proposed procedure included the steps of dispersion of the filler and polymer in an excess amount of solvent followed by solvent evaporation. In such a process the content of CNTs in TPU in the final material can be easily controlled by the weight ratio of the CNTs and polymer dispersed in the solvent. The proposed methods enabled the manufacture of the composites with 8 times higher CNTs content as compared to previously reported CNT/TPU composite fibers and amounted to 40 wt%. The maximum CNT content in the composite was determined based on mechanical characterization of a fiber formed out of the base composite material by hot extrusion process and feasibility of a fiber formation using this method.

The 40% *w*/*w* CNT fiber was characterized by very high electrical conductivity of 671 S/m which is over an order of magnitude higher than previously reported CNT/TPU fibers. Decreasing the CNTs content to 20% *w*/*w* decreased its conductivity by 2 orders of magnitude which is still a very good result as compared to previous materials. Moreover, both these materials showed a very high maximum current at failure of over 1 A and very high maximum elongations observed in the stress-strain curves. The mechanical testing showed also that the change of the CNTs content gives control over the stress and strain relation in the fibers.

All the presented results have shown that the proposed method can enable successful, simple and inexpensive production of highly conductive composites for various applications. It has been shown for example that, when considering smart-textile applications, such a fiber can be sewn into the fabric or easily transferred by ironing. However, considering both conductivity and elasticity of the material, and the fact that it can be easily formed thermally, its applications may be also sought among structural electronics applications, 3D printing techniques and molding technology.
